# Analysis of ethoxyquin and its oxidation products in swine tissues by gas chromatography-tandem mass spectrometry for evaluating the feed-to-animal tissue transfer of ethoxyquin and its metabolites

**DOI:** 10.1186/s40104-020-00529-z

**Published:** 2021-01-15

**Authors:** Chi Zhang, Xiangrong Gai, Ying Tian, Jiayi Wang, Dongting He, Wenjun Yang, Liying Zhang, Yiqiang Chen

**Affiliations:** State Key Laboratory of Animal Nutrition, College of Animal Science and Technology, China Agricultural University, Beijing, 100193 China

**Keywords:** Ethoxyquin, Ethoxyquin dimer, Ethoxyquin quinone imine, Gas chromatography-tandem mass spectrometry, Safety evaluation, Swine tissues

## Abstract

**Background:**

Ethoxyquin (EQ) is a common antioxidant which is widely used in animal feed. But the supplement of EQ in animal feed may lead to the residues of EQ and its major oxidation products: ethoxyquin quinone imine (EQI) and ethoxyquin dimer (EQDM) in animal tissue. Thus, it would pose potential health hazards to consumers. However, the method for the simultaneous determination of EQ, EQI and EQDM in animal tissues is currently not available, and the accumulation extend of these chemicals in animal tissues after EQ administration remains to be evaluated.

**Results:**

A gas chromatography-tandem mass spectrometry method was successfully developed for the simultaneous determination of EQ, EQI and EQDM in swine tissues. The quantitative limits of EQ, EQI and EQDM can achieve to 0.5, 5.0 and 5.0 μg/kg in swine tissues, respectively. The spiked-recovery ratios of the three analytes (5–2000 μg/kg) were in the range of 64.7%–100.7% with relative standard deviations below 11.6%. Moreover, the utilization of this method for the analysis of actual swine tissue samples revealed that the application of commercial EQ additive in swine diet would produce the residues of all the three chemicals (EQ, EQI and EQDM) in fat, kidney, liver and muscle.

**Conclusions:**

The assay accuracy and precision of this GC-MS/MS method can meet the requirement of quantitative analysis. Meanwhile, the safety of EQ as a feed additive should be seriously considered with regard to food safety concerns since the oxidation product of EQ may have potential carcinogenicity.

**Supplementary Information:**

The online version contains supplementary material available at 10.1186/s40104-020-00529-z.

## Background

Ethoxyquin (EQ) is one of the most potent antioxidants which is widely used in animal feed. It can effectively prevent the oxidation of fat and protein component in animal feed during storage [1]. Meanwhile, it can also preserve vitamin A and vitamin E in animal feed by preventing the formation of peroxides [1]. However, the supplement of EQ in animal feed can lead to its residue in animal tissue and would pose potential health hazards to consumers [2]. Thus, the US Food and Drug Administration (FDA) [3] has stipulated that the EQ limit is set at 150 mg/kg for feed and 0.5 mg/kg for animal muscle. Moreover, the European Commission [4] has temporarily forbidden the supplement of EQ in animal feed partly because the lack of data about the EQ metabolism and related toxicological studies. At present, the safety evaluation of EQ in animal feed and animal product is still underway, and thus more scientific information about the absorption, distribution, metabolism and excretion (ADME) of EQ in animal is required [5, 6]. Previous studies have indicated that the EQ may be oxidized into ethoxyquin dimer (EQDM) and ethoxyquin quinone imine (EQI) in the application process [7, 8]. The toxicological profile of the EQDM is considered similar to that of the EQ, while the EQI shows structural alerts for mutagenicity, carcinogenicity and DNA binding and thus it should be more seriously considered [9]. Consequently, the monitoring of EQDM and EQI as well as its precursor EQ residues in animal-origin food is quite necessary for the protection of consumer health [1, 10]. More importantly, the accumulation extent of EQ and its oxidation products in animal tissues following EQ application remains to be evaluated.

Currently, the methods for the determination of EQ mainly include thin layer chromatography [11], high performance liquid chromatography (HPLC) [12–15], gas chromatography (GC) and liquid chromatography-tandem mass spectrometry (LC-MS/MS) [16]. The thin layer chromatography is cumbersome, time-consuming and not sensitive. It is mainly used for the analysis of EQ content in oil due to the large interference error [11]. HPLC and GC coupled with fluorescence detector, electrochemical detector or mass spectrometry have been widely used for the accurate determination of EQ. For example, Aoki et al. [14] developed a high performance liquid chromatography-fluorescence detection for EQ in animal-origin food with limit of detection (LOD) of 10 ng/g, while Rodríguez-Gómez et al. [15] developed a liquid chromatography-electrochemical detection for EQ in aquatic products with LOD of 5 ng/g. Moreover, several LC-MS/MS methods have been developed for the determination of EQ and its metabolites in aquatic animal tissues [17, 18]. In this study, a GC coupled with tandem mass spectrometry (GC-MS/MS) method was firstly developed to simultaneously determine the residual amount of EQ, EQI and EQDM in swine tissues. Furthermore, this method was then applied to the analysis of these target chemicals in swine tissues following EQ application, aiming to evaluate the safety of EQ as a feed additive with regard to the animal-origin food safety.

## Methods

### Chemicals and apparatus

Acetone (analytical grade), n-hexane (analytical grade), and acetonitrile (HPLC grade) were all purchased from Beijing Chemical Reagent Company (Beijing, China). The EQ additive was provided by the Jiangsu Zhongdan Group Co., Ltd. (Taixing, China). The purity of the EQ additive was more than 95.0%, with a maximum of 0.001% lead, a maximum of 0.0002% arsenic and a maximum of 1% p-phenetidine. EQ, EQI and EQDM standards were all purchased from Sigma-Aldrich (St. Louis, MO., USA).

All sample analyses were carried out on an Agilent 7890A gas chromatography coupled with an Agilent 7000 tandem mass spectrometer system (Agilent Technologies, USA). The chromatographic separation was achieved on a gas chromatography capillary column (DB-WAX UI, Agilent J&W Scientific, USA). Drying operation was performed with a nitrogen blow concentrator (Beijing Kanglin Technology Co., Ltd., China).

### Instrumental conditions

The conditions of GC-MS/MS analysis were as follows: the inlet temperature was set at 250 °C, the injection volume of sample solution was 1 μL with split-less mode, and the flow rate of carrier gas was set at 0.9 mL/min. The oven temperature program was set at 100 °C for 1 min, followed by a 20 °C/min ramp to 240 °C and maintaining at 240 °C for 45 min. After GC separation, the analytes were detected by a triple quadrupole mass spectrometer equipped with an electron ionization (EI) source. The following parameters was employed: the interface temperature was set at 250 °C, the source temperature was set at 230 °C, the quadrupole temperature was set at 150 °C, the collision energy was set at 100 eV. The multiple reaction monitoring (MRM) mode was selected for the collection of MS signal. The precursor ions were selected as m/z 202, m/z 174, m/z 201, and the product ions were selected as m/z 174, m/z 130 and m/z 173 for quantitative analysis of EQ, EQI and EQDM, respectively. An Agilent MassHunter qualitative analysis software was used for data acquisition and processing.

### Sample preparation

Two grams of homogenized samples were weighed (accurate to 0.01 g) into a 50-mL stoppered centrifuge tube. Then 100 mg of ascorbic acid and 5 mL of sodium carbonate solution were sequentially added into the tube and gently mixed with the sample for 2 min with a vortex mixer. Subsequently, 5 mL of acetone was added and the sample solution was shaken for 2 min. Afterwards, 10 mL of n-hexane was added for liquid-liquid extraction by shaking for 2 min on a vortex mixer followed by centrifugation at 6000 r/min for 3 min. The upper layer was then transferred into a 50-mL centrifuge tube. The extraction was repeated twice and the n-hexane layer was combined in the centrifuge tube. Then it was dried in a 30 °C water bath with a nitrogen blow concentrator. The dried residue was reconstituted with 1.0 mL of acetonitrile and the re-dissolved solution was then vortexed for 1 min and sonicated for 2 min. Finally, the solution was filtered and subjected to GC-MS/MS analysis.

### Animal experiment

All animal handling and care procedures in these studies followed the specifications outlined by the Guide for the Care and Use of Agricultural Animals in Research and Teaching, and were approved by the Institutional Animal Care and Use Committee at China Agricultural University (CAU20160321-19). One hundred and eighty DLY (Duroc × Landrace ×Yorkshire) pigs with a body weight of 31.98 ± 2.34 kg were selected. The pigs were randomly divided into five treatments, with 6 replicates per treatment and 6 pigs per replicates. The dietary treatments were corn-soybean meal-based diets supplemented with 0, 150, 300, 750, 1500 mg/kg EQ. The experiment period was 98 days. The basic dietary nutrient level was formulated referring to NRC nutritional requirement of pig [19]. The experiment was conducted from December 2016 to April 2017 at the Animal Test Base of Ministry of Agriculture Feed Industry Centre (Fengning, Hebei). Before the experiment, the pig house was thoroughly disinfected, and the troughs and water tanks were cleaned. The pigs were routinely immunized. The temperature of the piggery was controlled at about 21 °C. Feed (mash form) and water (nipple drinker) were available ad libitum throughout the 98-d feeding trial. On the 98th day of the experiment, six pigs from six different replicates of each treatment were randomly selected and slaughtered on an empty stomach for 24 h. Before slaughtering, pigs were stunned with electric shock for 3 s and then sacrificed and bleed within 15 s according to the requirements of animal welfare. The dorsal longissimus muscle, liver, kidneys and abdominal fat were taken and collected at about 100 g and stored at − 20 °C. The contents of EQ and its main oxidation products in the above tissues were determined by the developed GC-MS/MS method.

### Method validation

The developed method was validated according to the Codex guideline (CAC/GL-71) [20, 21]. The limit of detection (LOD), limit of quantitation (LOQ), linearity, accuracy and precision were evaluated, respectively. The limit of detection (LOD) and limit of quantfication (LOQ) were calculated as the concentrations corresponding to three times and ten times peak areas (signal) as compared to chromatographic peak areas from blank sample (noise) [15, 20]. The linearity was assessed by the calibration curves of EQ, EQDM and EQI, which were constructed by plotting the peak areas of the three target analytes against the corresponding concentrations of the three analytes and fitted by linear mode. The method accuracy and precision were assessed by spiked recovery experiment. Blank swine muscle, kidney, liver and fat samples were obtained from pigs without feeding EQ in diets and were confirmed to be free of the three target chemicals by GC-MS/MS. Then the EQ, EQI and EQDM standard solutions were spiked into the blank samples to produce different spiked concentrations. The spiked samples were then treated and analyzed by GC-MS/MS as described above. The recovery ratio was calculated by comparing the measured concentration with the spiked concentration. For each spiked concentration, six replicate samples were measured in the same day to assess the intra-day precision, and three batches of samples were measured in three successive days to assess the inter-day precision.

## Results and discussion

### Optimization of GC-MS/MS conditions

In previous studies, several HPLC or GC methods have been developed for the determination of EQ by different research groups [12–18]. In this study, we utilized the GC-MS/MS for the determination of the EQ and its oxidation products EQDM and EQI, aiming to further improve the detection sensitivity and specificity. First, the mass spectra of EQ, EQDM and EQI under electron ionization were obtained using precursor ion scan mode of the mass spectrometry. As shown in Figure S1, the m/z 202.1, m/z 174.1 and m/z 200.9 can produce the highest signal under EI source (70 eV), thus they were selected as precursor ions for EQ, EQI and EQDM, respectively. Then, the collision energy from 0– 60 eV was optimized to obtain suitable product ions for each target chemical. The results indicated that the m/z 174.2, m/z 131.1 and m/z 173.1 corresponding to EQ, EQI and EQDM can produce the highest signals under product ion scan mode of the mass spectrometry. Therefore, these precursor ions and product ions were selected as the ion pairs in the tandem mass spectrometry to quantitatively determine the three target chemicals.

**Fig. 1:**
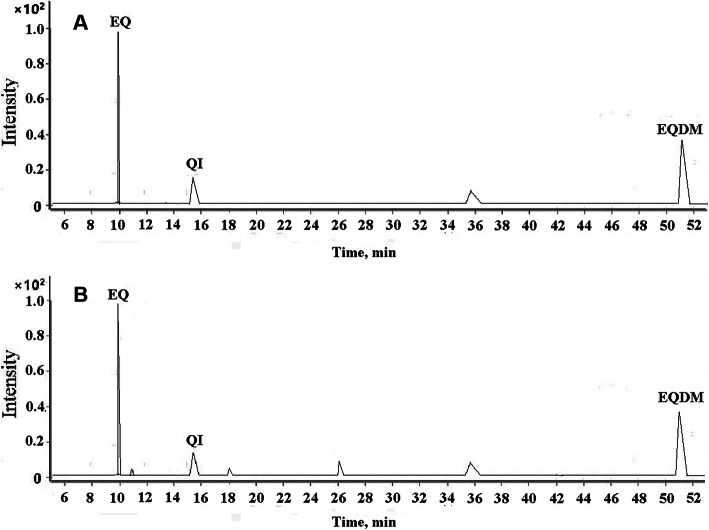
Typical MRM chromatograms of EQ, EQI and EQDM in buffer (**a**) and in sample solution (**b**)

Gas chromatography conditions, especially the heating program, play an important role in achieving the best separation of target chemicals. Therefore, the optimization of the heating program was performed to improve the separation of EQ, EQI and EQDM and the peak shape. As shown in Figure S2, the second programmed temperature procedure can achieve the best separation of the three chemicals with interferents from sample matrix, it was selected in the further experiment. Based on the optimized conditions, the representative MRM chromatograms is shown in Fig. 1, the retention time, quantitative ion pair, and collision energy parameters for EQ, EQI and EQDM are listed in Table S1.

### Optimization of sample preparation

**Fig. 2:**
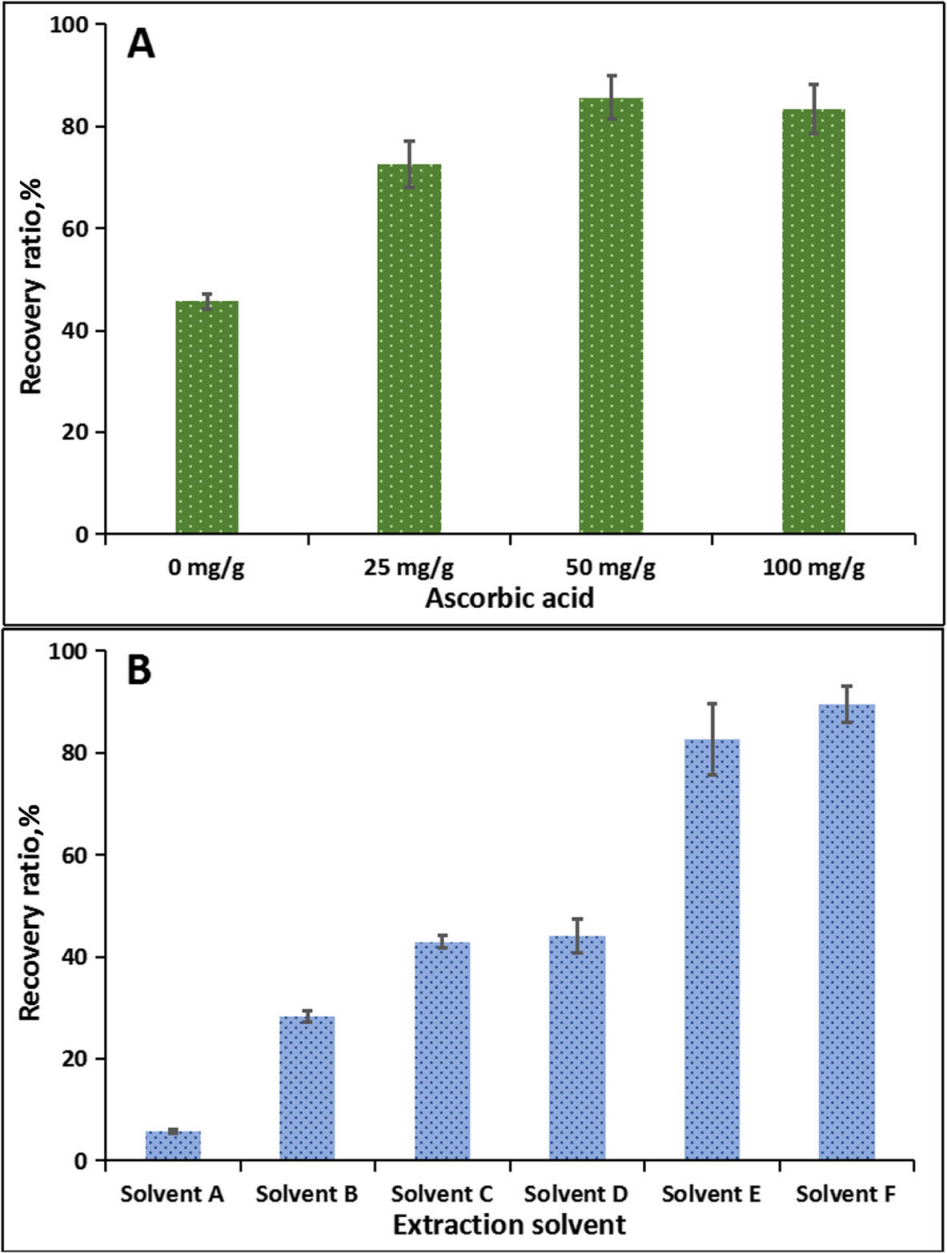
Effects of different concentrations of ascorbic acid and different extracting solvents on the extraction efficiency of EQ from swine tissues (*n* = 6). Note: Solvent A: acetonitrile; Solvent B: acetone; Solvent C: hexane; Solvent D: hexane+sodium sulphate; Solvent E: hexane+sodium carbonate; Solvent F: hexane+acetone+sodium carbonate

As an antioxidant, EQ can prevent the oxidation of other substances. In the meantime, it can be easily oxidized in the sample preparation process and thus would lead to analytical error especially at low level detection [22]. Thus, it is recommended to supplement some reductive substances to prevent or reduce the oxidation loss of EQ during sample preparation. Ascorbic acid is a typical reductive chemical and it is low-cost and easily available, therefore, it was selected to prevent the oxidation of EQ in this study. Three different levels of ascorbic acid (25, 50 and 100 mg/g sample) were tested to evaluate the effect of ascorbic acid supplementation on the recovery ratios of EQ at the spiked concentration of 100 μg/kg. As shown in Fig. 2a, compared to control treatment, the supplement of 25 mg ascorbic acid for 1 gram sample significantly improved the spiked recovery ratio from 44.6% to 78.5%. The addition of 50 mg ascorbic acid can further increase the recovery ratio to 95.4% and the addition of 100 mg ascorbic acid can not further increase this parameter. Therefore, 50 mg of ascorbic acid was added into each 1 gram of sample in the following experiment.

As EQ and its oxidation products are all low-polar chemicals, they are sparingly soluble in aqueous buffer but are soluble in some organic solvents. Thus, three different organic solvents including acetone, acetonitrile, n-hexane were firstly tested for the extraction efficiency. Unfortunately, the recovery ratios of ethoxyquin in sample by the three solvents were only 5.87% (acetonitrile) to 37.7% (n-hexane). The low extract efficiency by organic solvent may be attributed to the following reason: EQ and its analogs all contains a quinoline ring which includes a secondary amine or quaternary amine on the structure [2]. These amine groups can accept protons and serve as a weak organic base. Consequently, ethoxyquin-related molecules in animal tissues may be in the form of positive ions and thus they are hard to be directly extracted by organic solvent [9]. With an attempt to improve the extraction efficiency, sodium sulfate solution as a salting reagent was used prior to n-hexane extraction. However, the recovery ratio of EQ was still as low as 39.3%. Then the extraction protocol using carbonate buffer coupled with n-hexane was performed according to the previous studies. The use of carbonate buffer is supposed to inhibit the ionization of amine groups in the ethoxyquin structure, which can then facilitate the liquid-liquid extraction by n-hexane. The result indicated that this protocol is quite efficient and the recovery ratio of EQ from sample can reach 82.5%. As aqueous carbonate buffer and n-hexane are mutually insoluble, a part of EQ molecules may be lost because of insufficient liquid-liquid partition. Since acetone can be mutually soluble with both carbonate buffer and n-hexane, it was used to help the sufficient extraction of EQ by n-hexane. As expected, the protocol sequentially using carbonate buffer, acetone and n-hexane extraction further increased the recovery ratios of EQ from sample to 92.6% (Fig. 2b). Consequently, this protocol was final employed in the study.

### Method validation

#### Linearity

A serial diluted standard solutions of EQ, EQDM and EQI were analyzed by the developed GC-MS/MS method. Then three calibration curves were constructed by plotting the peak areas of the three target analytes against the corresponding concentrations of the three analytes. As shown in Table 1, at 0.5 to 100 ng/mL of EQ and 5.0 to 100 ng/mL of EQI and EQDM, the calibration curves were fitted well with linear mode with all the R^2^ more than 0.99.

**Table 1:** The linearity parameters of the calibration curves for EQ, EQI and EQDM

Chemicals	Linear range	Linear equation	R^**2**^
EQ	0.5–100 ng/mL	y = 762.2x-6.862	0.9998
EQI	5–100 ng/mL	y = 58.6x + 75.68	0.9976
EQDM	5–100 ng/mL	y = 93.31x-89.93	0.9999

#### LODs and LOQs

The LOD and LOQ were calculated as the concentrations corresponding to three times and ten times peak areas (signal) as compared to chromatographic peak areas from blank sample (noise) [15, 20]. By calculation, the LODs were 0.15 μg/kg, 1.5 μg/kg, and 1.5 μg/kg for EQ, EQI and EQDM, respectively and the LOQs were 0.5 μg/kg, 5.0 μg/kg, and 5.0 μg/kg for EQ, EQI and EQDM, respectively. These LODs and LOQs were below or comparable to that of other reported methods [12–18].

#### Assay accuracy and precision

For evaluating the assay accuracy and precision, a spiked-recovery experiment was performed. As shown in Table 2, at the spiked concentration of 5–2000 μg/kg, the spiked recovery ratios of EQ, EQI and EQDM from swine muscle, kidney, liver and fat were in the range of 64.7% –100.7%, with intra-day relative standard deviation (RSD) less than 7.54% (*n* = 6) and inter-day RSD less than 11.6% (*n* = 3). These results indicated that the assay accuracy and precision can basically meet the requirement for quantitative analysis and thus the developed GC-MS/MS method can be used for the analysis of real swine tissue samples.

**Table 2:** Intra-day and inter-day recovery ratios and relative standard deviation (RSD) of EQ, EQI and EQDM from swine tissues

Analyte	Samples	Spiked concentration, ng/g	Intra-day (*n* = 6)		Inter-day (*n* = 3)	
			Mean recovery, %	RSD, %	Mean recovery, %	RSD, %
EQ	Muscle	5	85.3	2.69	82.7	1.83
		25	95.9	1.83	87.5	0.82
	Kidney	100	91.0	2.99	78.2	4.00
		200	93.1	3.59	87.2	2.46
	Liver	100	89.8	5.7	82.9	4.06
		200	96.8	4.02	97.9	6.65
	Fat	1000	93.2	5.52	88.3	2.35
		2000	94.2	3.69	92.1	5.28
EQI	Muscle	5	74.3	1.71	68.7	2.64
		25	76.2	2.57	72.7	5.75
	Kidney	100	61.4	1.02	64.7	1.17
		200	68.8	3.11	70.2	7.81
	Liver	100	93.9	7.54	87.4	11.60
		200	89.2	4.21	82.6	6.78
	Fat	1000	79.3	5.61	82.3	4.63
		2000	81.2	4.76	75.5	5.12
EQDM	Muscle	5	83.3	1.51	82.3	1.53
		25	93.3	1.14	92.5	5.95
	Kidney	100	88.5	3.62	86.5	1.46
		200	99.2	2.29	93.0	6.73
	Liver	100	88.1	4.16	95.8	7.73
		200	100.7	2.51	89.5	4.74
	Fat	1000	77.6	4.81	74.2	5.11
		2000	82.5	6.42	84.7	5.79

### Analysis of EQ, EQI and EQDM residue in swine tissues

**Fig. 3:**
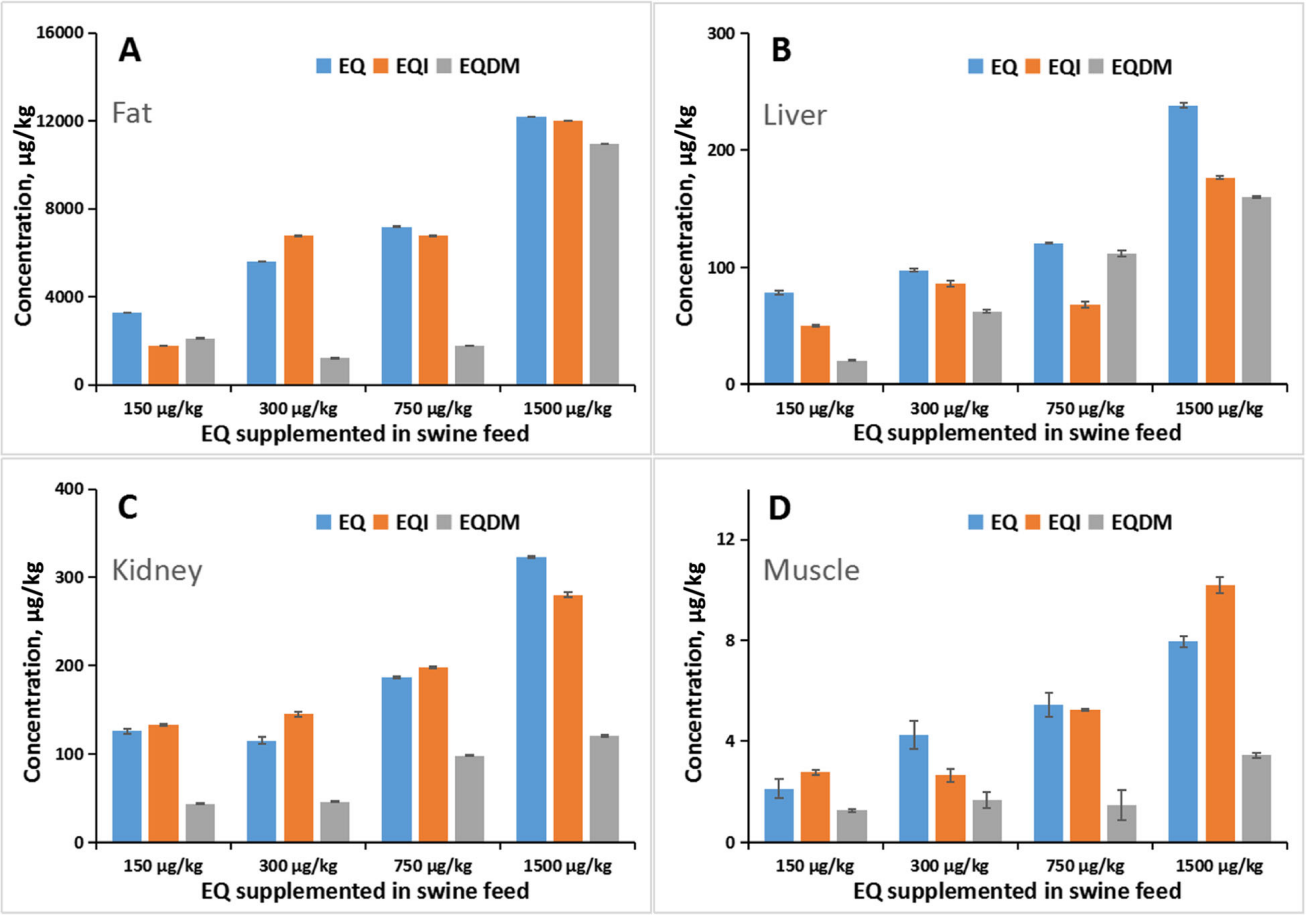
The residue amount of EQ, EQI and EQDM in fat (**a**), liver (**b**), kidney (**c**) and muscle (**d**) of of swine fed with different levels of EQ in diet

Currently, EQ is allowed to be used as an animal feed additive in China and the recommendation level is 150 mg/kg diet. In this study, pigs were fed with four treatment levels at the recommendation level, two times of recommendation level, five times of recommendation level and ten times of recommendation level, respectively. At the end of experiment (the 98^th^ day), swine muscle, liver, kidney and fat samples were collected and then analyzed by the developed GC-MS/MS method. As shown in Table S2, all the three chemicals (EQ, EQI and EQDM) were detected in all the samples. For the four treatments, the concentrations of EQ, EQI and EQDM in the fat samples were in the range of 3281–12193, 1780–12071 and 2112–10969 μg/kg, respectively; the concentrations of EQ, EQI and EQDM in the liver samples were in the range of 78.3–238, 50.2–177 and 20.1–160 μg/kg, respectively; the concentrations of EQ, EQI and EQDM in the kidney samples were in the range of 115–323, 133–280 and 43.5–121 μg/kg, respectively; and the concentrations of EQ, EQI and EQDM in the muscle samples were in the range of 2.12–7.95, 2.78–10.2 and 1.25–3.45 μg/kg, respectively. The results indicated that with the increased supplemented EQ level in diet, the concentrations of EQ, EQI and EQDM in all the swine tissues were all non-linearly elevated (Fig. 3). It demonstrated that the developed GC-MS/MS method can be used for actual sample analysis. On the other hand, the concentrations of all the three chemicals were the highest in fat sample and the lowest in muscle sample (Table S2 and Fig. 3). The extremely high concentration of EQ (3281–12193 μg/kg) and its oxidation product (1780–12071 μg/kg for EQI and 2112–10969 μg/kg for EQDM) in fat can be attributed to the fat-soluble characteristic of these chemicals. At the recommended level (150 mg/kg) of EQ in animal feed, the EQ level in fat is about 3281 μg/kg, which was below the tolerance set by US FDA [3]. But as the EQDM has similar toxicological profile with EQ [2], the sum of EQ and EQDM levels in fat would exceed the tolerance. Moreover, as swine oil is more often consumed by Chinese consumers than the consumers in Western countries, the tolerance of EQ level in fat may be required to be modified when considering the combined toxicities of EQ and EQDM. On the other hand, the concentration of EQ, EQI and EQDM in the muscle samples were below 7.95, 10.2 and 3.45 μg/kg, respectively, and the concentration of EQ, EQI and EQDM in the liver samples were below 238, 177 and 160 μg/kg, respectively (Table S2). The residue levels of EQ and EQDM in muscle and liver for the four treatments were all far below than the tolerances (0.5 mg/kg in muscle and 3 mg/kg in liver) set by the US FDA [3]. It seemingly suggests that the application of EQ as feed additive would have no potential hazards for consuming swine meat and liver, even considering the residue of EQDM [2]. However, the fact that all the swine tissues were found to contain EQI residues should be seriously considered. As the EQI may have mutagenicity and carcinogenicity, theoretically, its residue in animal-origin should be “zero” tolerable. In this regard, the application of EQ as a feed additive should be further evaluated due to its carry-over to animal product and undefined toxicological effect of its oxidation product EQI.

## Conclusion

In this study, a reliable GC-MS/MS method was developed and validated for the quantitative determination of EQ, EQI and EQDM in swine tissues. This method demonstrated high sensitivity, good selectivity and acceptable accuracy and precision, and it can be used as a routine tool for monitoring the residues of EQ and its oxidation products in swine tissues. Furthermore, the utilization of this method for actual swine tissue samples revealed that the application of commercial EQ additive in swine diet would produce the residues of all the three chemicals (EQ, EQI and EQDM) in fat, kidney, liver and muscle. Especially, the results suggest that the consumption of swine oil would be potentially hazardous if the swine was fed with EQ in the diet. Although the residue of EQ in swine muscle and liver would not result in health concerns to consumers, the fact that all the tissues contains EQI residue suggests the safety of EQ as an animal feed should be further evaluated. Further studies should be especially focused on the potential mutagenicity and carcinogenicity of EQI.

## Supplementary Information

**Additional file 1: Figure S1.** Structures, precursor ions and production ions of EQ and its oxidation products EQI and EQDM. **Figure S2.** The MRM chromatograms of EQ, EQI and EQDM using three different programmed temperature. **Table S1.** Detection parameters of EQ and its main oxidation products. **Table S2**. The determined concentrations of EQ, EQI and EQDM in tissues of swine fed with different levels of EQ in diet.

## Data Availability

All data generated or analyzed during this study are included in this published article.

## Abbreviations

EQ: Ethoxyquin
EQI: Ethoxyquin quinone imine
EQDM: Ethoxyquin dimer
EFSA: European Food Safety Authority
FDA: Food and Drug Administration
HPLC: High performance liquid chromatography
GC: Gas chromatography
GC-MS/MS: Gas chromatography coupled with tandem mass spectrometry
LOD: Limit of detection
LOQ: Limit of quantification
