# Supplementary material for: Analysis of ethoxyquin and its oxidation products in swine tissues by gas chromatography-tandem mass spectrometry for evaluating the feed-to-animal tissue transfer of ethoxyquin and its metabolites
Source: J Anim Sci Biotechnol. 2021 Jan 15;12:8. doi: 10.1186/s40104-020-00529-z (PMC7809739; doi:10.1186/s40104-020-00529-z)
Supplement: Supplementary file 1 — Additional file 1: Figure S1. Structures, precursor ions and production ions of EQ and its oxidation products EQI and EQDM. Figure S2. The MRM chromatograms of EQ, EQI and EQDM using three different programmed temperature. Table S1. Detection parameters of EQ and its main oxidation products. Table S2. The determined concentrations of EQ, EQI and EQDM in tissues of swine fed with different levels of EQ in diet. [file 40104_2020_529_MOESM1_ESM.docx]

Supplemental material

**Analysis of ethoxyquin and its oxidation products in swine tissues by gas chromatography-tandem mass spectrometry for evaluating the feed-to-animal tissue transfer of ethoxyquin and its metabolites**

Chi Zhang, Xiangrong Gai, Ying Tian, Jiayi Wang, Dongting He, Wenjun Yang, Liying Zhang *, Yiqiang Chen *

*State Key Laboratory of Animal Nutrition, College of Animal Science and Technology, China Agricultural University, Beijing 100193, China*

**^*^ Corresponding author:**

Liying Zhang, Ph. D, Professor

Tel: (86-10)62733764; Fax: (86-10)62733588; E-mail: [zhangliying01@sina.com](mailto:zhangliying01@sina.com)

Yiqiang Chen, Ph. D, Associate Professor

Tel: (86-10)62733764; Fax: (86-10)62733588; E-mail: [yqchen@cau.edu.cn](mailto:yqchen@cau.edu.cn)


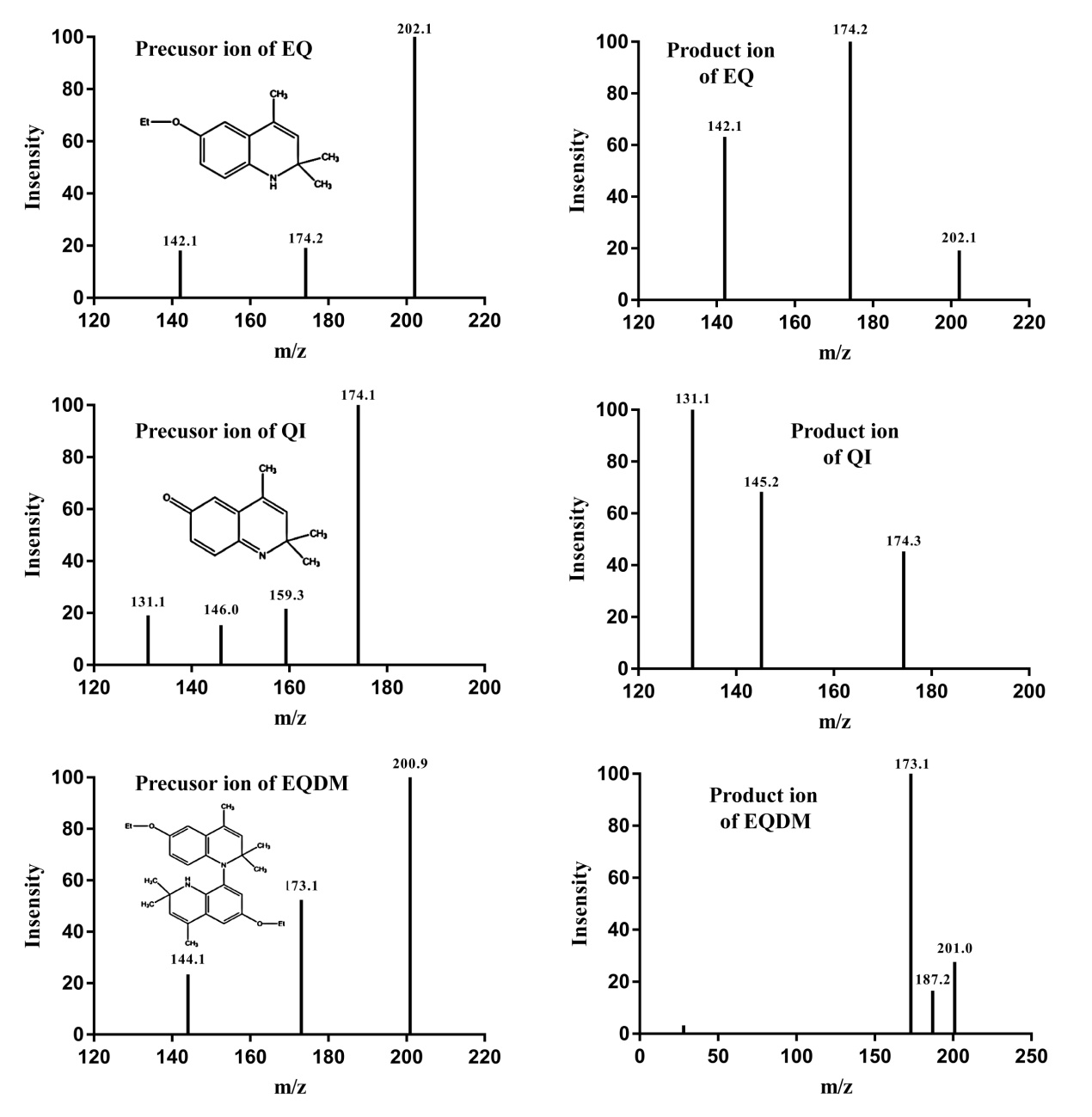


**Fig. S1** Structures, precursor ions and production ions of EQ and its oxidation products EQI and EQDM.


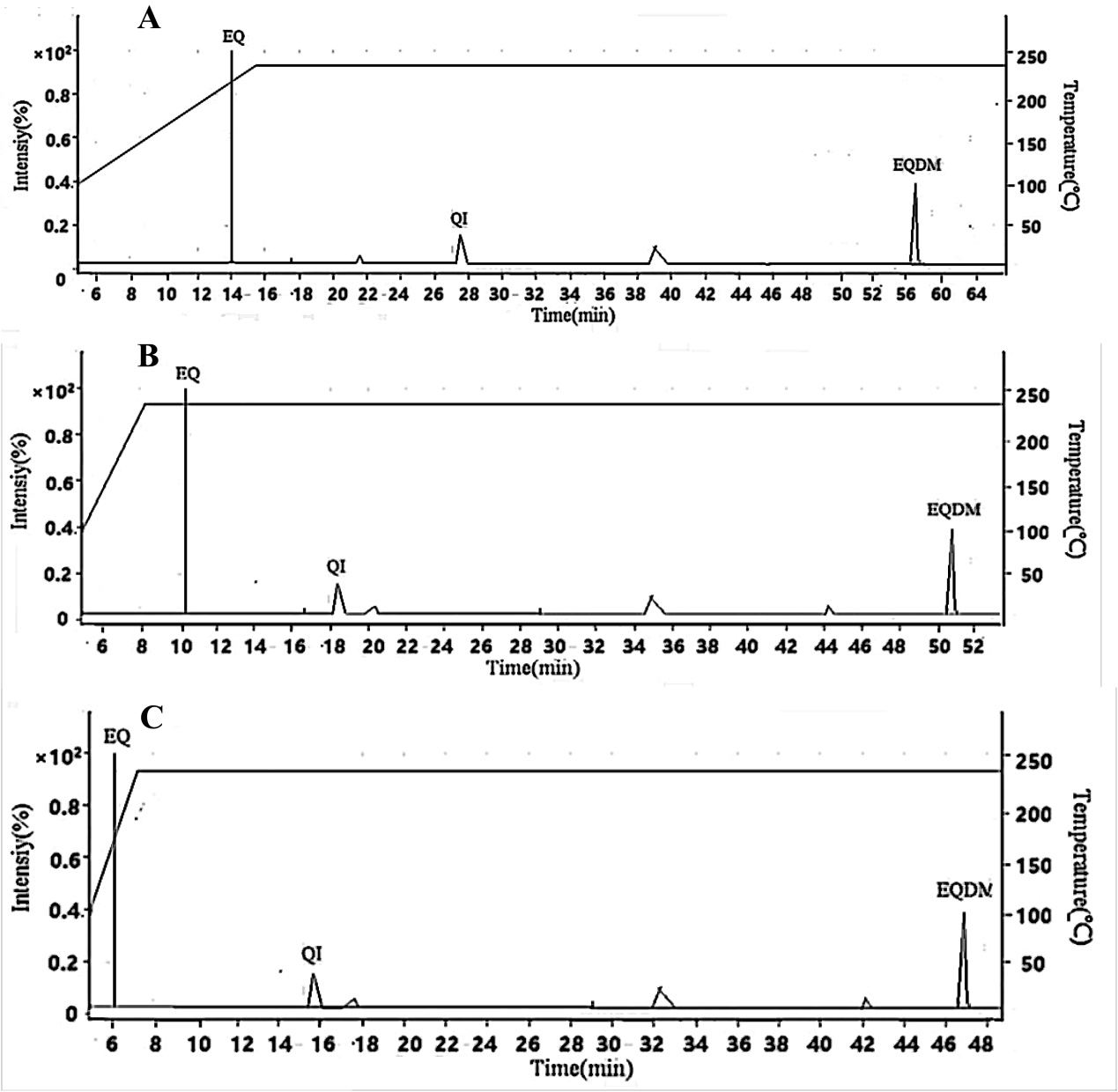


**Fig. S2** The MRM chromatograms of EQ, EQI and EQDM using three different programmed temperature.

**Table S1** Detection parameters of EQ and its main oxidation products

| Analyte | Retention time, min | Quantitative ion pair | |  | Qualitative ion pair | |
| --- | --- | --- | --- | --- | --- | --- |
|  |  | m/z | CE, eV |  | m/z | CE, eV |
| EQ | 9.985 | 202/174 | 100 |  | 202/142 | 100 |
| EQI | 18.426 | 174/130 | 100 |  | 174/145 | 100 |
| EQDM | 51.462 | 201/173 | 100 |  | 201/144 | 100 |

**Table S2** The determined concentrations of EQ, EQI and EQDM in tissues of swine fed with different levels of EQ in diet.

| Supplemented EQ in feed, mg/kg | Target chemicals in fat, μg/kg | | |  | Target chemicals in liver, μg/kg | | |  | Target chemicals in kidney, μg/kg | | |  | Target chemicals in muscle, μg/kg | | |
| --- | --- | --- | --- | --- | --- | --- | --- | --- | --- | --- | --- | --- | --- | --- | --- |
|  | EQ | EQI | EQDM |  | EQ | EQI | EQDM |  | EQ | EQI | EQDM |  | EQ | EQI | EQDM |
| 0 | N.D | N.D | N.D |  | N.D | N.D | N.D |  | N.D | N.D | N.D |  | N.D | N.D | N.D |
| 150 | 3281 | 1780 | 2111 |  | 78.3 | 50.2 | 20.1 |  | 126 | 133 | 43.5 |  | 2.12 | 2.78 | 1.25 |
| 300 | 5595 | 6783 | 1204 |  | 97.7 | 86.3 | 62.4 |  | 115 | 145 | 45.7 |  | 4.26 | 2.65 | 1.67 |
| 750 | 7183 | 6779 | 1771 |  | 121 | 68.2 | 111.8 |  | 187 | 198 | 98.2 |  | 5.44 | 5.26 | 1.46 |
| 1500 | 12194 | 12008 | 10969 |  | 238 | 177 | 160 |  | 323 | 280 | 121 |  | 7.95 | 10.2 | 3.45 |
